# Navigation in a Space With Moving Objects: Rats Can Avoid Specific Locations Defined With Respect to a Moving Robot

**DOI:** 10.3389/fnbeh.2020.576350

**Published:** 2020-11-12

**Authors:** Nikhil Ahuja, Veronika Lobellová, Aleš Stuchlík, Eduard Kelemen

**Affiliations:** ^1^Institute of Physiology, Czech Academy of Sciences, Prague, Czechia; ^2^Faculty of Science, Charles University, Prague, Czechia; ^3^National Institute of Mental Health, Klecany, Czechia

**Keywords:** navigation, dynamic environment, moving object, robot, hippocampus, place cells

## Abstract

Animals can organize their behavior with respect to other moving animals or objects; when hunting or escaping a predator, when migrating in groups or during various social interactions. In rats, we aimed to characterize spatial behaviors relative to moving objects and to explore the cognitive mechanisms controlling these behaviors. Three groups of animals were trained to avoid a mild foot-shock delivered in one of three positions: either in front, on the left side, or on the right side of a moving robot. We showed the rats can recognize and avoid these specific areas. The avoidance behavior specific for the left or right side of the robot demonstrated animals not only react to “simple” stimuli such as increasing noise level or growing retinal image of an approaching object, but they process their spatial position relative to the object. Using an all-white robot without prominent visual patterns that would distinguish its different sides, we showed that the behavior does not depend on responses to prominent visual patterns, but that the rats can guide their navigation according to geometrical spatial relationship relative to the moving object. Rats’ competence for navigation in space defined by a moving object resembles navigation abilities in stationary space. Recording of hippocampal single unit activity during rat’s interaction with the robot proved feasibility of the task to uncover neuronal mechanism of this type of navigation.

## Introduction

Navigation relative to other moving animals or objects is a cognitive ability that is important for animals in many ethologically relevant situations. Avoiding a predator, pursuing prey, moving within a migrating group, or collaborating within a hunting pack are among the many situations where orienting relative to moving animals is crucial. Although clearly important for animals’ success and survival, little is known about the cognitive and neuronal mechanisms controlling this type of behavior and laboratory methods to study this type of spatial behavior are needed. Importantly, it is not merely the distance from other moving animals or objects, but the precise position relative to them that determines successful navigation. Being in front of or behind a predator (or even competing conspecifics) makes an important difference in determining the proper course of actions. Here, we have developed a novel qualitatively advanced version of a spatial task to study the ability of freely moving rats to determine their position (not only distance) relative to a moving robot. We quantitatively and qualitatively characterize rat’s spatial behavior relative to a moving object. Finally, using single unit recordings we provide evidence that this task is suitable for studying the neuronal mechanisms supporting this type of navigation.

While spatial navigation in stationary environments has been studied extensively, the study of navigation in dynamic environments with moving objects or animals is only now gaining momentum. Prior research has begun to characterize changes in animals’ spontaneous behaviors and spatial navigation in the presence of another animal (Dorfman et al., 2016) or a robot (Shi et al., 2013; del Angel Ortiz et al., 2016); in addition a robot has been used to guide rats’ movements (Gianelli et al., 2018). In order to reinforce spatial behavior, we and other teams have used tasks requiring avoidance of a moving rat (Telensky et al., 2009) or a robot (Telensky et al., 2011; Kim et al., 2015). In previous work, rats were trained to avoid a circular area centered on a robot (Telensky et al., 2011; Svoboda et al., 2017), the rat’s exact position relative to the robot was not important (only distance). In this case the robot served as a moving “beacon” and the navigation strategy required was a variant of “taxon strategy” (Reddish, 1999) – a strategy based on approaching or avoiding a prominent landmark. In our current study, the rat did not need to avoid a moving object *per se* but to recognize its own position relative to the moving object and avoid a specific *place* defined relative to the object (a shock zone located in front of, or to one of the sides of the robot). Here, the robot is not simply a beacon, but a central reference point within a spatial coordinate system – a map that contains the shock zone location. Thus, our task contains features of “locale navigation strategy” – map-based navigation that typically does not rely on a single landmark but relies on constellation of complex spatial cues (Reddish, 1999).

In Experiments 1 and 2 we trained three groups of rats to move around a circular arena (130 cm in diameter) and avoid a mild foot shock delivered in a shock zone (39 cm in diameter) situated in one of three locations: either in front of, on the left side, or on the right side of a moving robot. Each rat was trained to avoid one shock zone location only. Experiment 1 comprised a version of the task with the shock zone in front of the robot which is ethologically more natural; it is the position in front of a predator, or a potentially aggressive conspecific that tends to be most dangerous in natural situations. Avoidance of an area in the front of an approaching object can be achieved by avoiding simple stimuli such as increasing noise level or increasing size of robot’s retinal image, without knowing spatial location relative to (in the reference frame of) the robot. To assess more advanced, spatial avoidance strategy, we used a version of the task with the shock zone on one side of the robot in Experiment 2. Successful avoidance behavior in this task cannot rely solely on increasing noise level or increasing retinal size of approaching object, because the shock zone on one side of the robot and the safe zone on the opposite side do not differ in these parameters. The importance of assessing the rat’s own position relative to the robot is thus enhanced. Comparing the number of entrances to (or time spent in) the shock zone to the opposite safe zone is thus an optimal parameter to assess avoidance behavior in Experiment 2. In Experiment 1, with the shock zone in front of the robot, there is not an obvious single ‘mirror image’ control zone to use for comparison. Therefore, to quantify avoidance behavior in this task, we compared the number of entrances to the shock zone to the mean of the three safe zones (one on each side, and one behind the robot).

**FIGURE 1:**
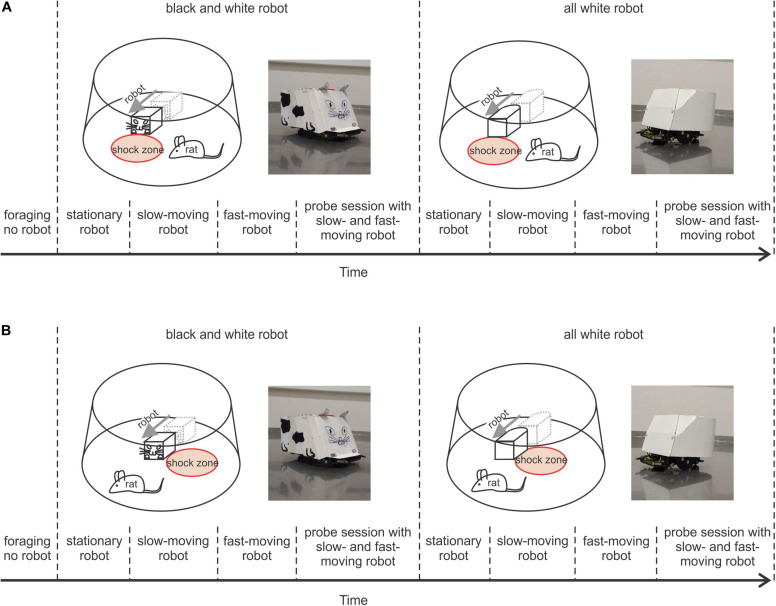
Behavioral protocols. **(A)** Protocol of Experiment 1, where the rat avoided a shock zone in front of the robot. **Upper left** plot shows a schematic of the arena, a rat, and black and white (B&W) robot with the shock zone at the front. To the right is a photograph of the B&W robot; **upper right**, shows a schematic of the arena, rat, and all-white robot with the shock zone at the front, and a photograph of the all-white robot. The lower part depicts the timeline of the experiment. **(B)** Protocol of Experiment 2, where a rat avoided the shock zone on one side of the robot. Plots are organized in a way analogous to **A**.

To explore the importance of prominent visual cues in this type of navigation, we used two versions of robot design. Each rat was first trained in a version of the task with the robot painted like a stylized cat when each side had a distinct visual look (black and white robot – B&W – Figure 1A, left). Then we used an all-white robot (Figure 1A, right) to reduce visual patterns that could serve as cues, forcing the rat to rely on the distinct geometry of the rounded back and “boxy” front of the robot to guide avoidance behavior. Under these conditions, the importance of relying more on the spatial relationship relative to the robot was enhanced.

## Materials and Methods

### Animals

Male Long-Evans rats aged three months (*N* = 19) from the breeding colony of the Institute of Physiology, Czech Academy of Sciences, Prague were used. Each animal was housed in a separate cage in a temperature-controlled room (22 ± 2°C) with a 12 h light/12 h dark cycle (lights on at 6:00 am). The experiments were performed in light phase of the day. Water was freely available. Diet was restricted to maintain the rats at ≥ 80**%** of their free-feeding weight. All animal procedures were approved by the Committee for the Ethical Treatment of Animals and Animal Welfare at the Institute of Physiology, Czech Academy of Sciences and by the departmental committee of the Czech Academy of Sciences (Project of Experiments No. 136/2013), and complied with the Animal Protection Act of the Czechia and European Union directive 2010/63/EC.

### Experimental Set-Up

Prior to the experiment, a miniature connector was attached to the skull of each rat using bone screws and dental cement under isoflurane anesthesia. The connector was used to attach colored marks (blue and red) for tracking the rat’s position and head direction during behavioral testing. Similar marks of different colors (orange and yellow) were used to track the position and orientation of the robot.

**FIGURE 2:**
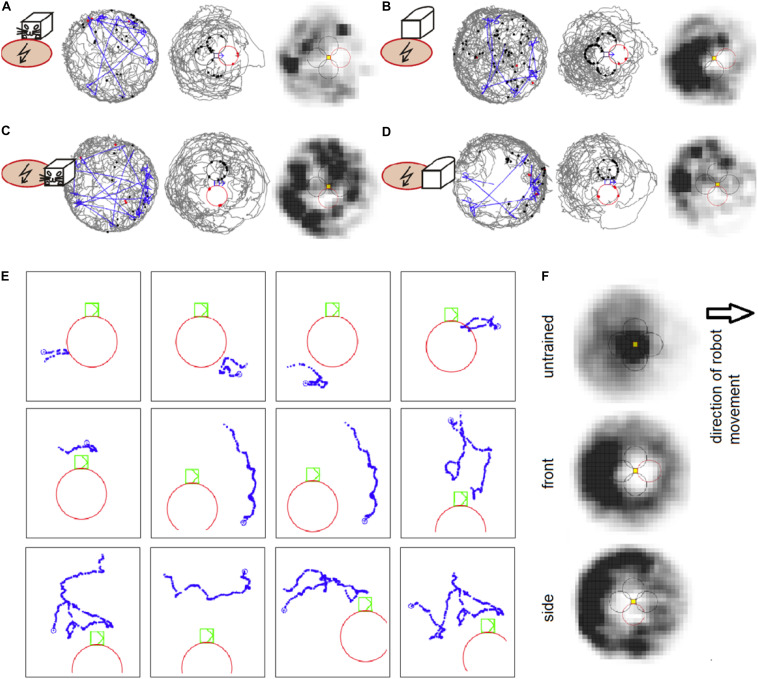
**(A)** An example session of avoidance of the shock zone in front of the B&W robot. The left plot shows the trajectory of a rat (gray) and the robot (blue) on the arena. The middle plot shows the trajectory of a rat relative to the robot, which is approximately in the middle of the plot. The shock zone is shown by a red circle, other equidistant safe zones are marked by black circles. Red dots indicate points of rat’s entrance to the shock zone and black dots indicate points of entrance to the safe zones. The right plot depicts the time spent by the rat in different locations relative to the robot. Darker colors mark more visited locations. **(B)** An example session with the shock zone in front of the all-white robot. **(C)** Example session with the shock zone on the right side of the B&W robot. **(D)** Example session with the shock zone on the right side of the all-white robot. **(E)** Examples of rat’s behavior when close to the robot during the session shown in **D**. Twelve example trajectories when the rat was within 20 cm of the shock zone (at the right side of the robot) or equidistant “safe” zones around the robot are shown. Robot is shown in green color, rat’s trajectory in blue, and the shock zone in red. **(F)** Average heat maps in control sessions of untrained rats that interacted with the robot and during the probe sessions in rats trained to avoid front and right side of the robot.

The experiments were performed in a small square room (2.5 m × 2.5 m). The window of the experimental room was darkened, so the room was without natural light and was lit by two tube lights housed in louver fixture, used for typical indoor office lighting. The behavioral experiments were performed on an elevated circular arena (130 cm in diameter) with a transparent plastic wall (50 cm high). The rats were trained to avoid a circular shock zone (39 cm in diameter) defined by its position relative to a moving Arduino-programmed robot (16 cm long, 12 cm wide, and 10.5 cm tall). The rats were randomly divided into three groups according to the position of the shock zone: the shock zone was located either (1) in front of the robot (Figure 1A), or (2) on the left or (3) right side of the robot (Figure 1B). The robot was programmed to move in close to linear trajectories (resembling an arc with a large diameter) until it hit the wall of the arena; then it moved backward 10 cm, waited for 15 s, turned at a random angle between 100° and 200° and continued moving forward. Custom-made software (Kachna tracker, author Tomáš Mládek) tracked the position and orientation of both the rat and the robot. Whenever the rat entered the shock zone, it received a mild foot-shock (0.2–0.5 mA, constant current, 50 Hz) lasting 500 milliseconds and repeated after 400 milliseconds until departure from the shock zone. The current level was adjusted for each rat between 0.2–0.5 mA to the lowest level that elicited avoidance behavior. For most of the animals the shock amplitude was set at 0.2 mA for all reinforced sessions. Shock was delivered between two electrodes. One electrode consisted of a cable hanging from above the arena and connected via an alligator clip to a piercing ring (made of a syringe needle) attached to the rat’s skin between shoulders. The metal surface of the arena served as the other electrode (Telensky et al., 2009, 2011). The impact of the electric current was localized to the point of the greatest resistance between the paws and the arena. Two robot designs were used: in the first design, the robot was painted white with a black drawing of a stylized cat face at the front, tail at the back and two legs on each side (black and white robot – B&W – left side of Figures 1A,B). The second robot design was all-white (right side of Figures 1A,B). The rats were first trained with the B&W robot and subsequently with the all-white robot. The rats were trained gradually over successive sessions, first with the robot stationary, then slow-moving (2 cm/s) and finally, fast-moving (4 cm/s).

### Behavioral Training Procedure and Testing Protocol

In Experiment 1, a group of rats (*N* = 5) was trained to avoid a shock zone in front of the robot (Figure 1A), and in Experiment 2 another group of rats (*N* = 10) was trained to avoid left or right side of the robot (Figure 1B). The position of the shock zone (front, left or right) was fixed for each rat for all the experiments. Rats in both experiments were subjected to the same four-stage training and shaping protocol, which only differed in shock zone location.

#### Stage 1

Foraging without the robot: After a week of handling, rats were trained to forage for pasta pellets on the arena without the robot present. The pellets served to reinforce the rats to walk on the arena throughout all subsequent stages of the experiment. Rats had three 10-min foraging sessions per day with 10–15 min interval between the sessions and were trained until they walked ≥ 40 m per session in three subsequent sessions. The rats reached this criterion in 3–5 days.

#### Stage 2

Avoiding the stationary robot: The animals were trained to avoid a shock zone in three 10-min daily sessions with a stationary robot, with 10–15 min intersession intervals. The position of the robot was changed between sessions but did not change within a session. The animals were trained until they reached the criterion of ≤ 8 entrances to the shock zone per session across three consecutive days. The rats reached this criterion in one to 2 weeks with the B&W robot, and within 1 week with the all-white robot. See Supplementary Materials, Figure 1 for details.

#### Stage 3

Avoiding the slow-moving robot: The transition from stationary to slow-moving (2 cm/s) sessions was gradual. Of the three training sessions performed each day, the first two (later in training only the first one) were with the stationary robot and the last one (later, the last two) were with slow-moving robot. The animals were trained under these conditions until the criterion of ≤ 8 entrances to the shock zone per session across three consecutive days was reached. The rats reached this criterion in 1–5 weeks with the B&W robot, and within 2 weeks with the all-white robot. The criterion was reached faster by rats that had to avoid the front of the robot. After the criterion was met, two subsequent sessions with the slow-moving robot were used to characterize and statistically evaluate each animal’s performance, as is presented in the “Results” section.

#### Stage 4

Avoiding the fast-moving robot: During this stage, the rats were trained in three sessions a day: the first was stationary, the second was with the slow-moving robot, and the third with the fast-moving robot. By the end of the training with the fast-moving robot, the rats were undergoing one stationary, and two fast sessions each day. The rats were trained until they reached criterion of ≤ 8 entrances to the shock zone per session across three consecutive days. The rats reached this criterion in 1–5 weeks with the B&W robot, and within 2 weeks with all-white robot (see Supplementary Materials, Figure 1 for details). The criterion was reached faster in rats that had to avoid the front of the robot. Their performance was analyzed and statistically evaluated in two subsequent fast-moving sessions.

Some of the animals showed immobility or freezing during the first few days into each training stage. This behavior was present when the animals got their first few shocks with the stationary and moving robot, particularly in the fast condition. The freezing behavior waned off gradually with training as the rats learned to avoid the particular zone.

After the rats learned the task, their performance was tested in probe sessions that were performed in exactly the same way as the reinforced sessions except that shocks were not delivered. Probe sessions with the slow-moving and fast-moving robot were performed on different days, with at least 2 days of reinforced training in between.

**FIGURE 3:**
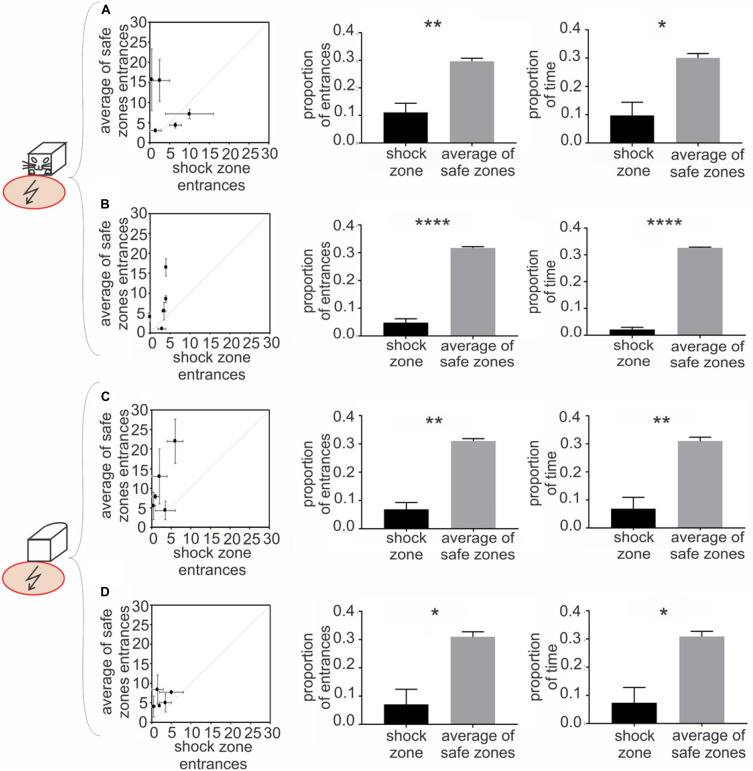
Experiment 1 – Avoidance of a shock zone in front of the robot. Data from slow-moving B&W robot **(A)**, fast-moving B&W robot **(B)**, slow-moving all-white robot **(C)** and fast-moving all-white robot **(D)** are shown. **Left** scatter plots show number of entrances to the shock zone versus mean number of entrances to the three safe zones, during two reinforced sessions after the training criteria was met for each of five rats (see Materials and Methods). **Central** figures quantify entrances to the shock zone and safe zones in unreinforced probe sessions. **Right** figures show time spent in the shock zone and safe zones in unreinforced probe sessions. Plots show means ± SEM. *p < 0.05, **p < 0.01, ****p < 0.0001.

**FIGURE 4:**
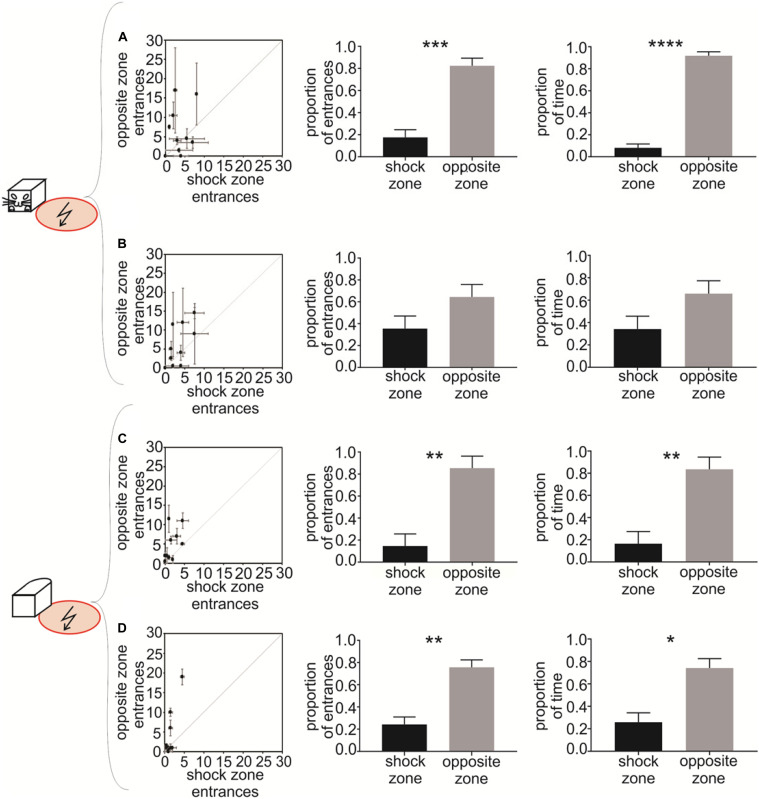
Experiment 2 – Avoidance of a shock zone at the side of the robot. Data from slow-moving B&W robot **(A)**, fast-moving B&W robot **(B)**, slow-moving all-white robot **(C)**, and fast-moving all-white robot **(D)** are shown. **Left** scatter plots show the number of entrances to the shock zone versus the number of entrances to the safe zone, during two reinforced sessions after the training criterion was met by each of the 10 rats. **Central** figures show the proportion of entrances to the shock zone and safe zone in unreinforced probe sessions. **Right** figures show the proportion of time spent in the shock zone and safe zone in unreinforced probe sessions. Plots show means ± SEM. *p < 0.05, **p < 0.01, ***p < 0.001, ****p < 0.0001.

After avoidance behavior was characterized in well-trained rats using the B&W robot (Figures 3A,B, 4A,B), we proceeded to assess whether avoidance depended on recognition of prominent visual patterns painted on the robot. With all-white robot we followed the same protocol as with the B&W robot (Figures 3C,D, 4C,D). We first trained the rats until they reached stable avoidance behavior and then performed probe sessions.

### Electrophysiology

Additional four rats were used in electrophysiological Experiment 3, where activity of hippocampal neurons was recorded while a rat interacted with the moving robot. This experiment was performed to show feasibility of the presented behavioral paradigm for investigation of the neuronal mechanism underlying navigation relative to the moving robot. Activity of hippocampal neurons was recorded using tetrode technique described previously (Kelemen and Fenton, 2010). Briefly, rats were anesthetized with isoflurane, skull was exposed, trephine opening was drilled to the skull, and 32 recording electrodes organized into eight independently movable tetrodes were implanted above the dorsal hippocampus (4.2 mm posterior, 2.0 mm lateral to Bregma). After recovery from the surgery, position of the tetrodes was adjusted to yield clearly distinct unit recordings. Hippocampal activity was recorded during the task on the arena with the robot by Cheetah recording system and Lynx8 amplifiers (Neuralynx, Bozeman, MT, United States) and analyzed by Spike 2 (CED, Cambridge, United Kingdom) and custom made programs in Matlab (MathWorks, Natick, MA, United States).

### Data Analysis

To characterize and quantify avoidance behavior in Experiment 1 (Figure 3), the number of the rat’s entrances to the shock zone in front of the robot was compared to the mean number of entrances to the three equidistant safe zones on sides of and behind the robot (Figures 2A,B). Proportion of entrances to the shock zone *P*(shock) was calculated as

$$P\,\left(\text{shock}\right)=\frac{N\,\left(\text{shock}\right)}{N\,\left(\text{shock}\right)+N\,\left(\text{safe}\right)}$$

Where *N*(shock) is the number of shock zone entrances and *N*(safe) is the number of entrances to all three safe zones. The mean proportion of entrances to the safe zones *P*(safe) was calculated as

$$P\,\left(\text{safe}\right)=\frac{N\,\left(\text{safe}\right)}{\left(N\,\left(\text{shock}\right)+N\,\left(\text{safe}\right)\right)*3}$$

Where *N*(shock) is the number of shock zone entrances and *N*(safe) is the number of entrances to all three safe zones. Analogous formulas were used to calculate the proportion of time spent in the shock zone and the proportion of time spent in safe zones during probe sessions (shown in the right column of Figure 3).

To characterize and quantify avoidance behavior in Experiment 2 (Figure 4), the number of the rat’s entrances to the shock zone on one side of the robot was compared to the number of entrances to the safe zone on the opposite side of the robot (Figures 2C,D). The proportion of entrances to the shock zone *P*(shock) was calculated as

$$P\,\left(\text{shock}\right)=\frac{N\,\left(\text{shock}\right)}{N\,\left(\text{shock}\right)+N\,\left(\text{opposite}\right)}$$

Where *N*(shock) is the number of shock zone entrances and *N*(opposite) is the number of entrances to the opposite safe zone. The proportion of entrances to the safe zone P(opposite) was calculated as

$$P\,\left(\text{opposite}\right)=\frac{N\,\left(\text{opposite}\right)}{N\,\left(\text{shock}\right)+N\,\left(\text{opposite}\right)}$$

Analogous formulas were used to calculate the proportion of time spent in the shock zone and proportion of time spent in the opposite safe zone during probe sessions (shown in the right column of Figure 4).

The data were analyzed using custom script written in MATLAB (MathWorks, MA, United States). For statistical analysis we used one-tailed, paired t-tests (GraphPad PRISM 7, San Diego, CA, United States). Statistical significance was tested at α = 0.05.

## Results

### Experiment 1

Experiment 1 showed that the rats avoided the shock zone in front of the moving robot. The performance of well-trained rats during example sessions avoiding the B&W robot and all-white robot is shown on Figures 2A,B, respectively. Compared to untrained rats, which spent time in the vicinity of the moving robot, the rats trained for avoidance behavior were spending less time around the robot and avoided the shock zone in particular, as can be seen in time-averaged dwell-time-maps (Figure 2F). More refined analyses were used for detailed characterization of the rats’ avoidance behavior.

The rats were trained to criterion of ≤ 8 entrances to the shock zone per session across three consecutive days. The performance of all five rats during the first two post-criterion sessions is depicted in the left column of Figure 3 for each of the four experimental conditions: (1) slow-moving (2 cm/sec) B&W robot (Figure 3A), (2) fast-moving (4 cm/sec) B&W robot (Figure 3B), (3) slow-moving all-white robot (Figure 3C), and (4) fast-moving all-white robot (Figure 3D). With progressive training, the number of shock zone entrances decreased to 2.5 ± 0.8, while the number of entrances to the safe zones remained at 5.8 ± 0.9 in two post-criterion sessions with the fast-moving all-white robot (Figure 3D). The animals’ tendency to enter the shock zone less than safe zones is shown by data points above the diagonal in plots in the left column of Figure 3.

Probe sessions, when avoidance was not reinforced by the foot shock, were used to further quantify avoidance of the front of the robot in Experiment 1. For the B&W slow-moving robot, both the proportion of entrances to the shock zone [*t*(4) = 4.167 *p* = 0.007, Figure 3A, middle] and time spent in the shock zone [*t*(4) = 3.264, *p* = 0.015, Figure 3A, right] were significantly smaller than in corresponding safe zones. Results were similar in the three other conditions: for the B&W fast-moving robot, the proportion of entrances to the shock zone [*t*(4) = 14.79, *p* < 0.0001, Figure 3B, middle] and time spent in the shock zone [*t*(4) = 28.99, *p* < 0.0001, Figure 3B, right] were also both significantly smaller than in the safe zones. For the all-white slow-moving robot, again, both the proportion of entrances to the shock zone [*t*(4) = 6.61, *p* = 0.0014, Figure 3C, middle] and time spent in the shock zone [*t*(4) = 4.277, *p* = 0.0065, Figure 3C, right] were significantly smaller than in corresponding safe zones. And finally, for the all-white fast-moving robot, the proportion of the shock zone entrances [*t*(4) = 3.332, *p* = 0.0145, Figure 3D, middle] and time in the shock zone [*t*(4) = 3.247, *p* = 0.016, Figure 3D, right] were also significantly smaller than in the safe zones.

Time to the first entrance to the shock zone and mean time of the first entrance to the safe zones was analyzed and compared next. If the animal did not enter a particular zone during the whole session, 600 s – corresponding to the duration of a session was scored. We observed consistent tendency of the rats to enter the shock zone later than the other zones. This tendency was significant for the fast moving all-white robot (*p* < 0.05, see Supplementary Materials, Figure 4 for details).

In probe trials we next detected each entrance to the shock zone and safe zones, and characterized each single entrance using following parameters: time spent in zone, average distance between a rat and the robot, and average speed of the rat. We did not detect systematic difference between shock zone entrances and safe zone entrances in any of these parameters (data not shown). This suggests that in spite of significantly lower numbers of shock zone entrances, once an animal made a mistake and entered the shock zone, its behavior there was not apparently distinct from behavior in safe zone.

To determine whether the movement of the robot was crucial for recognition and avoidance of the front side of the robot, we analyzed avoidance behavior in sessions with a stationary robot, which were performed on the same day as probe sessions with the moving robot. We observed that, in stationary sessions with the B&W robot, the proportion of entrances was significantly smaller in the shock zone compared to the mean of the safe zones [slow probe day: *t*(4) = 5.707, *p* = 0.0023; fast probe day: *t*(4) = 11.03, *p* = 0.0002]. Similarly, with the all-white robot, the proportion of entrances to the shock was significantly smaller compared with the mean of corresponding safe zones [slow probe day: *t*(4) = 9.708, *p* = 0.0003; fast probe day: *t*(4) = 36.91, *p* < 0.0001]. These results show that recognition and avoidance of the front of the robot is not contingent upon the robot’s movement (see Supplementary Materials, Figure 2).

### Experiment 2

Experiment 2 showed that the rats avoided the shock zone on the side of the moving robot. The performance of well-trained rats during example sessions avoiding the B&W robot and all-white robot is shown in Figures 2C,D, respectively. Rat’s behavior during single approaches (within 20 cm) of shock zone and safe zones is illustrated in Figure 2E. Twelve examples of single trajectories are shown. The first three panels in the top row show successful avoidance, when the rat approached the shock zone and turned around before entering it. The fourth panel shows an example of an entrance to the shock zone. The other panels show examples of the rat approaching the robot through the control safe areas.

To quantify the avoidance behavior in Experiment 2, the number of entrances to the shock zone on one side of the robot was compared to the number of entrances to the control safe zone on the opposite side of the robot. The performance of all 10 rats during two post-criterion sessions (see Materials and Methods) is depicted in the left column of Figure 4 for each of the four experimental conditions: (1) slow-moving B&W robot (Figure 4A), (2) fast-moving B&W robot (Figure 4B), (3) slow-moving all-white robot (Figure 4C), and (4) fast-moving all-white robot (Figure 4D). The animals’ tendency to enter the shock zone less than the safe zone on the opposite side is shown by data points above the diagonal in Figures 4A–D. With progressive training, the number of shock zone entrances decreased to 1.5 ± 0.4, while the number of entrances to the safe zone remained at 4.1 ± 1.9 in two post-criterion sessions with the fast-moving all-white robot (Figure 4D).

Probe sessions were used to further quantify avoidance of the side of the robot in Experiment 2. For the B&W slow-moving robot, both the proportion of entrances to the shock zone [*t*(8) = 4.696, *p* = 0.0008, Figure 4A, middle] and time spent in the shock zone [*t*(8) = 11.860, *p* < 0.0001, Figure 4A, right] were significantly smaller than in the corresponding opposite safe zone. In the case of the B&W fast-moving robot, the proportion of entrances to the shock zone [*t*(7) = 1.262, *p* = 0.124, Figure 4B, middle] and time spent in the shock zone [*t*(7) = 1.369, *p* = 0.107, Figure 4B, right] were also smaller than in the corresponding opposite safe zone, however, this was non-significant. For the all-white slow-moving robot, both the proportion of the shock zone entrances [*t*(8) = 3.222, *p* = 0.006, Figure 4C, middle] and time in the shock zone [*t*(8) = 3.044, *p* = 0.008, Figure 4C, right] were again significantly smaller than in the opposite safe zone. And finally, in the all-white fast-moving robot, again, both the proportion of the shock zone entrances [*t*(8) = 3.845, *p* = 0.003, Figure 4D, middle] and time in the shock zone [*t*(8) = 2.890, *p* = 0.01, Figure 4D, right] were significantly smaller than in the corresponding safe zone.

Time to the first entrance to the shock zone and time of the first entrance to the opposite safe zone was analyzed and compared next. We observed consistent tendency of the rats to enter the shock zone later than the safe zone. This tendency was significant for the slow moving B&W robot (*p* < 0.05) and slow moving all-white robot (*p* < 0.01, see Supplementary Materials, Figure 4 for details). We next detected each entrance to the shock zone and the opposite safe zone, and characterized each single entrance using time spent in zone, average distance between a rat and the robot, and speed of the rat. We did not detect systematic difference between shock zone entrances and safe zone entrances in any of these parameters (data not shown).

We next addressed the importance of robot movement for recognition of the left or right side of the robot. We analyzed robot avoidance behavior in sessions with a stationary robot, which were performed on the same day as probe sessions with moving robot. We observed that in stationary sessions with B&W robot the proportion of entrances in the shock zone was significantly smaller compared with the opposite safe zone [slow probe day: *t*(9) = 8.513, *p* < 0.0001; fast probe day: *t*(9) = 12.84, *p* < 0.0001]. Similarly, with all-white robot the proportion of entrances was significantly smaller in the shock zone [slow probe day: *t*(9) = 30.58, *p* < 0.0001; fast probe day: *t*(9) = 13.67, *p* < 0.0001]. These results show that recognition and avoidance of one side of the robot in Experiment 2 is not dependent on the robot’s movement (see Supplementary Materials, Figure 3 for details).

### Hippocampal Unit Activity

To prove feasibility and relevance of the presented task for study of neuronal mechanism of navigation relative to a moving object, we performed single cells neuronal recordings. We recorded 46 hippocampal CA1 complex spike cells (putative pyramidal neurons) from three rats in no-shock conditions and 50 cells from one rat trained for the robot avoidance task.

**FIGURE 5:**
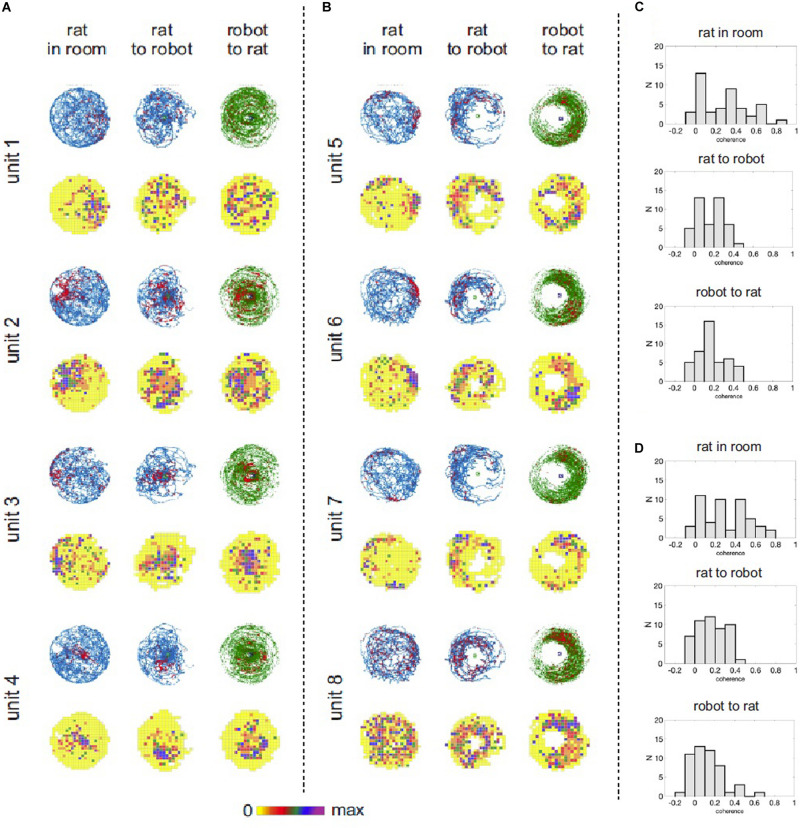
Hippocampal neuronal responses during rat navigation with a moving robot. **(A)** Spatial activity of four example hippocampal units recorded from untrained rats with a fast moving robot. Neuronal spatial activity is shown in three frames of reference: (1) rat’s position in the room, (2) rat’s position relative to the robot, (3) robot’s position relative to the rat. For each unit the **upper row** shows position and spike maps. The rat’s position is depicted in blue, robot’s position is shown in green. Locations of action potential discharge are shown by red dots. The **lower row** shows firing rate maps, where mean firing rate at each visited pixel is color coded – from yellow (0 spikes/sec) to purple – maximal firing rate for a particular neuron. **(B)** Spatial activity of four example hippocampal units recorded from trained rats avoiding right side of a fast moving robot. **(C)** Distribution of spatial coherence derived from firing rate maps in the three reference frames in untrained rats with fast moving robot. **(D)** Distribution of spatial coherence derived from firing rate maps in the three reference frames in the trained rat avoiding right side of a fast moving robot.

For each of 14 experimental recording sessions we characterized spatial activity of recorded *place cells* in three reference frames (coordinate systems): (a) position of a rat in the experimental room, (b) position of a rat relative to the robot, and (c) position of the robot relative to the rat. We used standard spike maps and rate maps to depict and assess spatial organization of neuronal activity in the task. Spike maps and rate maps indicated that activity of some neurons reflected different aspects of the interaction between the rat, the robot, and their environment (Figure 5). Some neurons had theirs action potential discharge organized according to a rat’s position in the room, but not according to rat’s position relative to the robot (e.g., units 1, 2, Figure 5). Other neurons appeared to be responding to the mutual position of a rat and the robot (units 3, 6, Figure 5). Yet other cells seemed to have firing organized conjunctly in multiple reference frames (unit 4, Figure 5) or disorganized (unit 8, Figure 5). We used parameters of spatial coherence (Muller and Kubie, 1989) and spatial information (Skaggs et al., 1993) to quantify spatial organization of neuronal activity in all three reference frames (coordinate systems) for all the recorded cells (Figure 5C,D). In the recordings from the trained rat, ANOVA revealed significant effect of spatial reference frame on spatial coherence [*F*(2,94) = 29.72; *p* < 0.0001]. Tukey *post hoc* test showed higher spatial coherence values for rat-in-room coordinate system than for rat-to-robot and robot-to-rat coordinate systems (*p*’s < 0.0001). In untrained rats, a similar effect of spatial reference frame on spatial coherence was observed [*F*(2,86) = 8.727; *p* = 0.0004]. Tukey *post hoc* test showed higher spatial coherence values for rat-in-room coordinate system than for rat-to-robot and robot-to-rat coordinate systems (*p*’s < 0.001).

For the trained rat, ANOVA revealed significant effect of spatial reference frame on information content [*F*(2,94) = 5.611; *p* = 0.005]. Tukey *post hoc* test showed higher information values for rat-in-room coordinate system than for rat-to-robot (*p* = 0.0040) but no significant difference between values in rat-in-room versus robot-to-rat coordinate systems (*p* = 0.52). For untrained rats, no significant difference was found in the information content across reference frames [*F*(2,86) = 0.08661; *p* = 0.9171].

## Discussion

An individual’s ability to assess its position relative to moving objects and animals, be it predators, prey or conspecifics, is cognitive competence that is important not only for basic survival in many animal species but also for social interactions. In a new behavioral paradigm, we showed that animals can recognize specific spatial positions that is not marked directly, but is defined by its spatial relationship in reference to a distal moving robot. Our behavioral paradigm can be understood in framework of instrumental conditioning, where stimulus leads to a response conditioned by negative consequences. It is the nature of the conditioned stimulus the rats are responding to, that is focus of our study. Experimental manipulations showed that the spatial avoidance behavior was robust and flexible, controlled by complex cues. In Experiment 1, we demonstrated that the rats learned to avoid the circular shock zone in front of the moving robot. In Experiment 2, we demonstrated the rats’ ability to avoid the shock zone on one side (left or right) of the moving robot. Using the all-white robot, we showed that the rats were able to perform the same tasks under the conditions when drawings on the robot could not be used as cues controlling avoidance behavior. This set of experiments showed that avoidance behavior is based not merely on recognizing prominent simple stimuli, such as increasing noise levels, the size of retinal image of an object or particular visual patterns characterizing the object. We conclude that the rats respond to a complex spatial stimulus that can be described using the framework of spatial coordinate system centered on the robot.

In Experiment 2, when the rats had to avoid the shock zone on the side of the fast-moving B&W robot, the tendency for avoidance was not statistically significant at α = 0.05 (Figure 4B) and was weaker than in Experiment 1 with the shock zone in front of the robot. We can speculate that this difference can be attributed to the fact that avoiding front of a dangerous object (animal or inanimate object) is ethologically more common situation than avoiding the side of it. It is also possible that it is easier to recognize the front of the robot from the other relatively distinct-looking sides, than it is to recognize the left side from the right, which look more similar. Nevertheless, after additional training the rats were able to avoid the side of the robot as accurately as the front, as was manifested in the subsequent avoidance behavior with the all-white robot (Figures 4C,D).

Previously, several experiments studied rat orientation and navigation with respect to moving objects, some of them originating in our laboratory. Pastalkova et al. (2003) demonstrated the ability of rats to assess the position of objects on a rotating, but inaccessible scene. Since in this work rats were restrained in a Skinner box, this experiment did not reveal whether the rats recognized the position of the moving object in allocentric space or the position of the moving object relative to the rat itself. These two options can be dissociated when a moving rat is observing another moving object. In a series of experiments, we showed that moving rats are able to keep a safe distance from a moving robot (Telensky et al., 2009), an ability that depends on intact hippocampus (Telensky et al., 2011) and intact anterior cingulate cortex (Svoboda et al., 2017). Our current work extends these previous findings by showing that the rat can assess not just the distance from a moving significant object, but also its own relative position to the object. Thus our current task is a logical next step toward modeling and understanding spatial behavior in complex dynamic environments.

While neural representation of stationary objects has been studied systematically in the hippocampus (Gothard et al., 1996; Rivard et al., 2004), anterior cingulate cortex (Weible et al., 2009, 2012), perirhinal cortex (Burke et al., 2012; Deshmukh et al., 2012; Burke and Barnes, 2015) and medial (Høydal et al., 2019) and lateral entorhinal cortex (Deshmukh and Knierim, 2011; Wang et al., 2018), the neuronal substrate for representations of moving objects were previously assessed in only a few studies. Earlier studies reported that hippocampal neurons responded to the position of moving objects in allocentric space but they rarely assessed the responses to position of a rat relative to moving objects (for such exception see Ho et al., 2008). In these studies, the position of moving objects (inanimate or animate) in space did not influence hippocampal place cells much (Zynyuk et al., 2012; Gianelli et al., 2018), modulated place cell firing a little (Ho et al., 2008), or was represented in the firing of hippocampal cells (Danjo et al., 2018; Omer et al., 2018). The likelihood of observing influence of moving objects on neuronal firing increased when the object’s position was more relevant for the rat in a particular experiment. Our hippocampal CA1 recordings showed that neuronal firing reflects various aspects of spatial interaction between a rat, a moving robot and their environment. We observed that neuronal activation patterns reflected position of a rat in the room, and mutual position between a rat and the robot. These data demonstrate that our behavioral task is a valid and useful model for further analysis of neuronal mechanisms of spatial behavior in relation to a moving object.

Theories of spatial navigation typically consider navigation in a single planar Cartesian reference frame with two dimensions (Reddish, 1999; but see Touretzky and Muller, 2006). In our task, processing the rat’s position relative to the robot is reinforced. In addition to this information, the rat could also process types of information defined in other frames of reference, i.e., information about rat’s position in the room, information about the robot’s position in the room, and about the robot’s position relative to the rat. Navigation in dynamic environments, such as environments enriched with moving objects presented in this paper, calls for extension of classical spatial navigation theories to include multiple reference frames of navigation. There are two basic (not mutually exclusive) ways how spatial information about distinct concurrently present and relevant frames of reference can be processed in the brain: one approach assumes different spatial frames are processed independently, the competing view suggests a single integrated complex representation of multiple spatial aspect of complex experience. Experimental findings pertinent to this topic remain so far inconclusive. In hippocampal recordings, neuronal ensembles representing distinct frames of reference were shown to be active at different times organized by a so-called functional grouping mechanism (Kelemen and Fenton, 2010, 2013, 2016; Jezek et al., 2011). There is also evidence for modulation of hippocampal spatial neuronal responses by additional parameters, such as animal’s vertical position, creating three dimensional representation (Hayman et al., 2011). Extension of this ability for multi-dimensional representations could provide an alternative means for representation of moving objects in an environment.

In the presented series of experiments, we used aversively motivated task, it is thus possible, that the robot was perceived as a “predator.” Of the defensive behaviors associated with response to a predator according to the predatory imminence theory (Bolles and Fanselow, 1980; Fanselow and Lester, 1988), we observed freezing and avoidance early in the training. With proceeding training the former behavior subsided and the latter prevailed consistent with our previous work (for example: Kelemen and Fenton, 2010, 2013; Svoboda et al., 2017). The mild shock used in the task likely triggered stress response in animals. Stress affects acquisition, consolidation and retrieval of memory in important ways (Kirschbaum et al., 1996; de Quervain et al., 2000, 2009; Maheu et al., 2005; Wolf, 2009; Jezek et al., 2010; Wilhelm et al., 2011; Rimmele et al., 2013; Kelemen et al., 2014). Stress hormones have particularly strong effect on hippocampus dependent memories (de Quervain et al., 2009; Wilhelm et al., 2011; Kelemen et al., 2014). Although the basic findings on organization of navigation relative to a moving object, described in this paper, likely apply also for appetitively motivated tasks, this topic can be further explored in subsequent appetitively motivated behavioral studies requiring “cooperation” with a moving partner rather than avoidance. Our presented work can also be extended to detect brain regions involved in our task, in addition to neurobiological or electrophysiological work to identify neuronal substrate of this type of navigation.

In summary, we behaviorally characterized and analyzed the rats’ ability to navigate with respect to a moving object. In our experiments the rats avoided specific position defined in relation to a moving robot, either front of the robot or one side of the robot. The avoidance did not depend on prominent visual patterns (drawings) on the robot, as the geometry of all-white robot was sufficient to guide the avoidance behavior. Robot movement was not necessary as the avoidance behavior was preserved also in the presence of stationary robot. This important and cognitively challenging aspect of navigation deserves further study on the level of behavior as well as underlying neuronal mechanisms required for coordination of own position and position of an object.

## Data Availability Statement

The raw data supporting the conclusions of this article will be made available by the authors, without undue reservation.

## Ethics Statement

The animal study was reviewed and approved by Committee for the Ethical Treatment of Animals and Animal Welfare at the Institute of Physiology, Czech Academy of Sciences and (Project of Experiments No. 50/2016) and complied with the Animal Protection Act of the Czech Republic and European Union directive 2010/63/EC.

## Author Contributions

NA, AS, and EK designed the study. NA and VL performed the experiments. NA and EK analyzed the data and wrote the manuscript. AS and EK provided supervision, scientific leadership, and funding acquisition. All authors reviewed the manuscript.

## Conflict of Interest

The authors declare that the research was conducted in the absence of any commercial or financial relationships that could be construed as a potential conflict of interest.
