# Supplementary material for: Navigation in a Space With Moving Objects: Rats Can Avoid Specific Locations Defined With Respect to a Moving Robot
Source: Front Behav Neurosci. 2020 Nov 12;14:576350. doi: 10.3389/fnbeh.2020.576350 (PMC7689095; doi:10.3389/fnbeh.2020.576350)
Supplement: Supplementary file 1 [file Table_1.DOCX]

|  | **Stationary** | **Slow** | **Fast** |
| --- | --- | --- | --- |
| Front avoidance/B&W robot | | | |
| **Average time to criterion (days)** | 8 | 14.2 | 8 |
| **SEM (days)** | 0.447214 | 4.673329 | 0.447214 |
|  |  |  |  |
| Front avoidance/all white robot | | | |
| **Average time to criterion (days)** | 3 | 4.8 | 5.4 |
| **SEM (days)** | 0 | 0.734847 | 1.122497 |
|  |  |  |  |
| Side avoidance/B&W robot | | | |
| **Average time to criterion (days)** | 8.7 | 16.9 | 12.2 |
| **SEM (days)** | 0.334996 | 2.77608 | 2.803965 |
|  |  |  |  |
| Side avoidance/all white robot | | | |
| **Average time to criterion (days)** | 4 | 7.4 | 7 |
| **SEM (days)** | 0.421637 | 0.669992 | 0.298142 |

**Table 1: Average training time. The table shows average time required to reach criterion for three phases of training, stationary, slow and fast along with the variation among animals (standard error of the mean, SEM).**

A


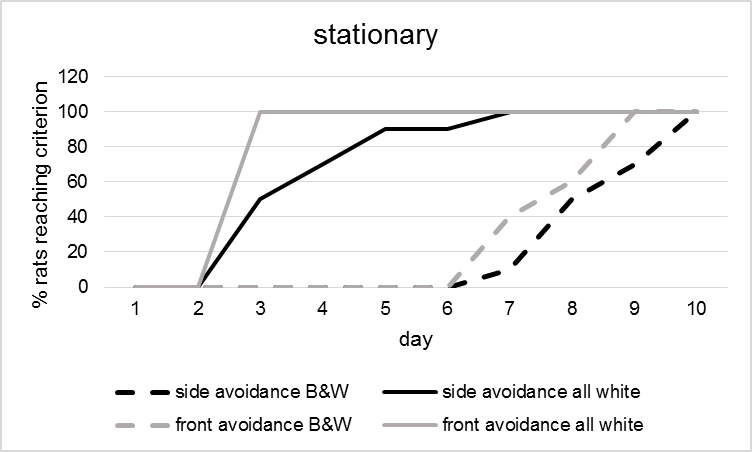


B


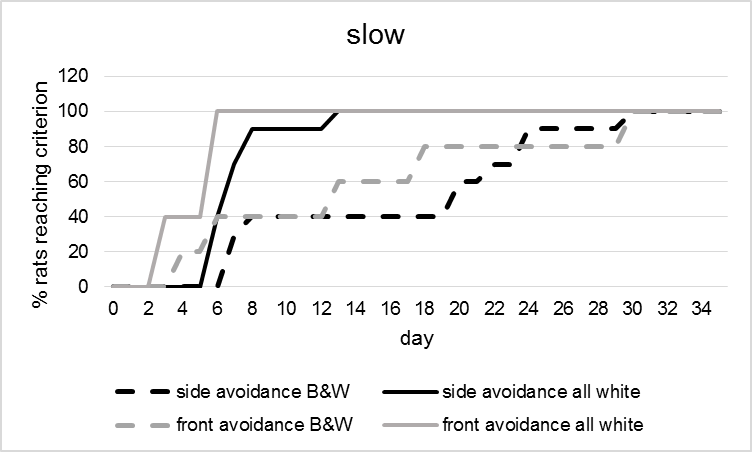


C


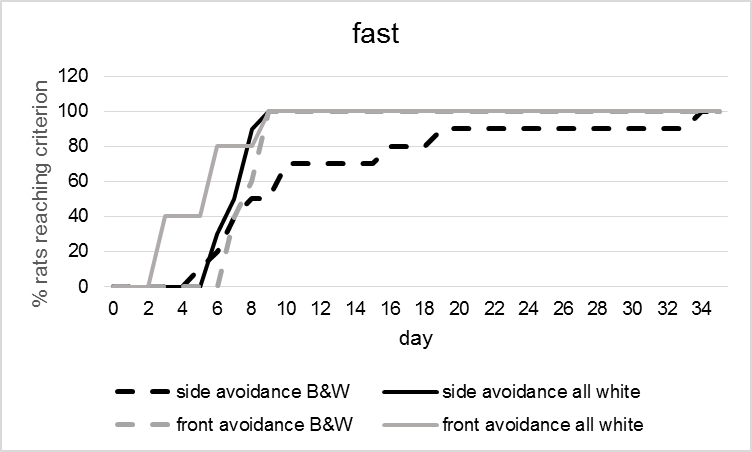


**Figure 1. Cumulative curves for each training condition and phase. (a), (b) and (c) show percentage of rats achieving the behavioral criterion on different days of training for stationary, slow and fast phases of the training.**


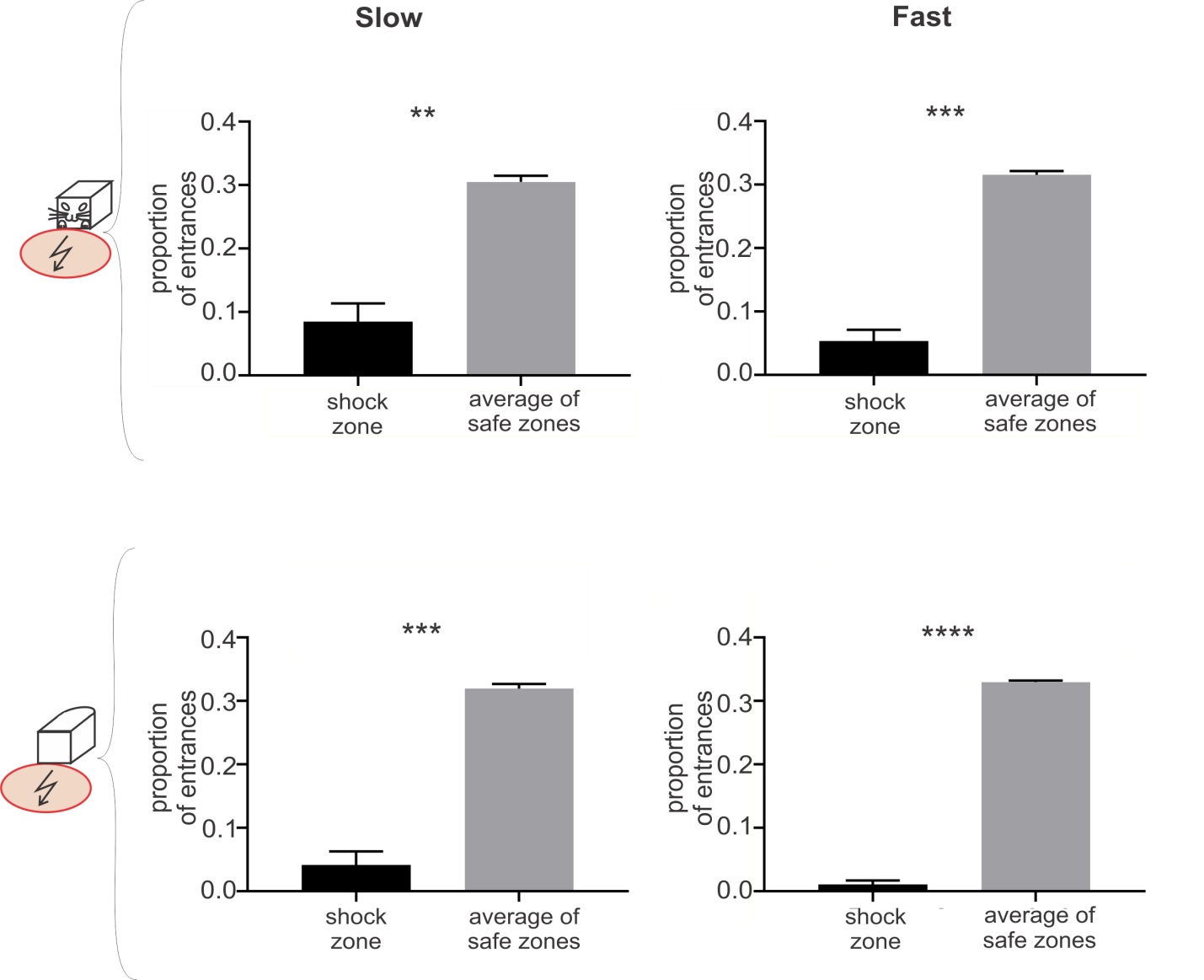


**Figure 2: Performance in stationary sessions on slow and fast probe trial days in experiment 1. Proportion of entrances in the shock versus average of safe zones for front avoidance is shown; upper left graph is from stationary session on slow probe trial day with B&W robot and the upper right graph is from fast probe trial day. Lower part follows similar pattern except that it was with all-white robot. Plots show means ± SEM. **p<0.01, ***p<0.001, ****p<0.0001.**


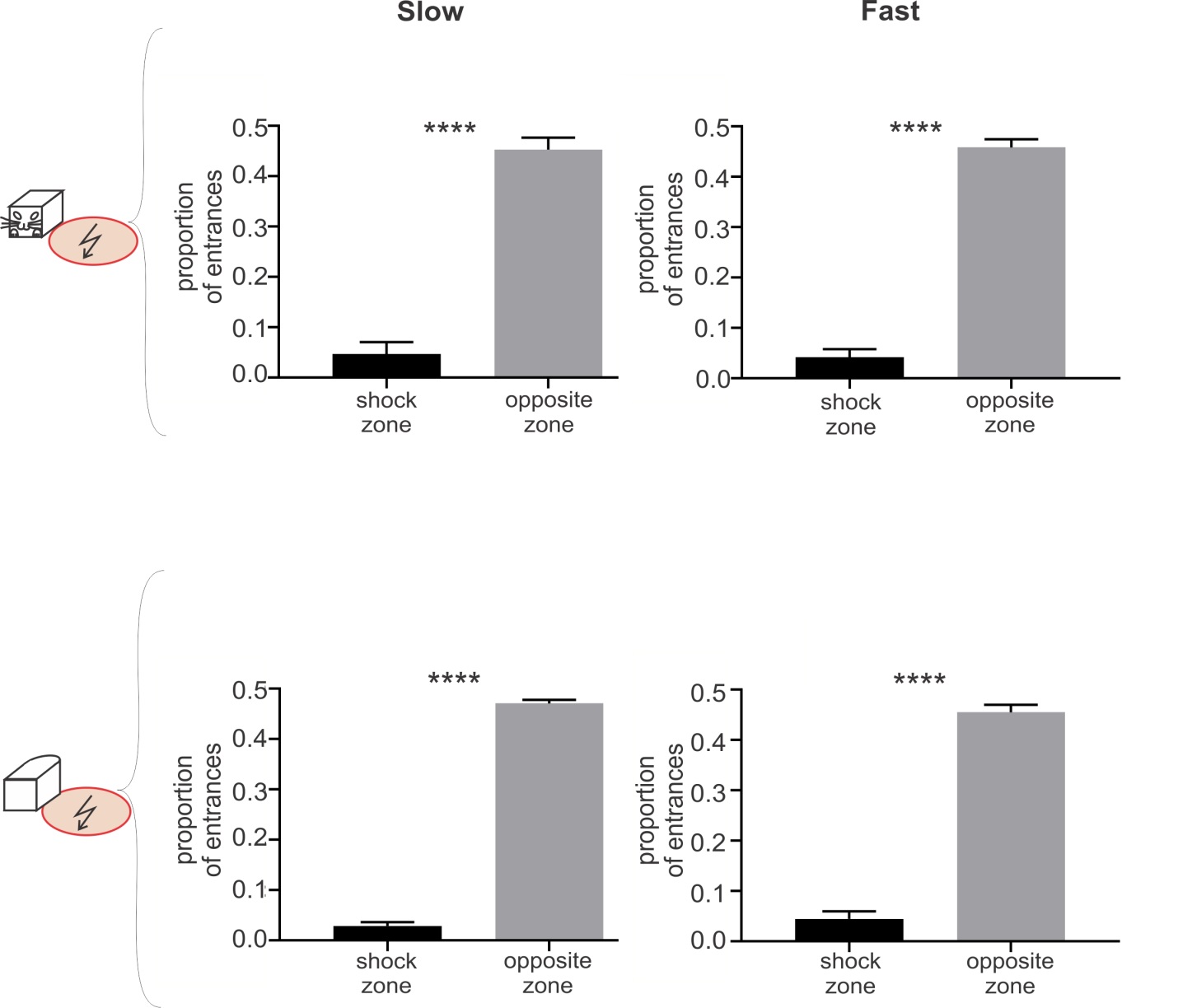


**Figure 3: Performance in stationary sessions on slow and fast probe trial days in experiment 2. Proportion of entrances in the shock versus opposite zone from side avoidance is shown. The upper left graph is from stationary session on slow probe trial day with B&W robot and the upper right is from fast probe trial day. Lower graphs are from respective probe trial days with all-white robot. Plots show means ± SEM. **p<0.01, ***p<0.001, ****p<0.0001.**
